# Longitudinal changes in pain catastrophizing and multidimensional health outcomes after passive-recharge burst spinal cord stimulation: a cohort study with follow-up to two years

**DOI:** 10.1007/s00701-026-07033-9

**Published:** 2026-09-15

**Authors:** Johan Wänman, Jakob Östman, Pedram Tabatabaei

**Affiliations:** 1 Department of Clinical Sciences, Neurosciences, Umeå University, 90185 Umeå, Sweden; 2 Department of Diagnostics and Intervention, Umeå University, 90731 Umeå, Sweden

**Keywords:** Spinal cord stimulation, Pain catastrophizing, Patient reported outcomes

## Abstract

**Purpose:**

To evaluate longitudinal changes in pain catastrophizing following spinal cord stimulation (SCS) and to determine whether baseline pain catastrophizing was associated with patient-reported outcomes over two years after implantation.

**Methods:**

A retrospective cohort study using prospectively collected data from 58 patients undergoing SCS implantation. Pain catastrophizing was assessed using the Pain Catastrophizing Scale (PCS), and pain intensity and multidimensional health outcomes were assessed using the Numeric Rating Scale (NRS) and PROMIS-29, respectively. The primary outcome was change in PCS from baseline. Analyses of PCS were restricted to patients with available baseline PCS data (*n* = 38). PCS was assessed at baseline and at 3, 6, 12, and 24 months after SCS implantation. Changes from baseline were evaluated using paired t-tests. Associations between baseline PCS and post-implant outcomes were assessed using Spearmans rank correlation.

**Results:**

Among the 38 patients with baseline PCS data, mean PCS scores decreased from 24 ± 12 at baseline to 14.0 ± 11 at 24 months (*p* < 0.001), with reductions across all three PCS subgroups. Physical function, pain interference, pain intensity, fatigue, anxiety, depression, and participation in social roles also improved significantly during follow-up, whereas sleep disturbance showed no significant change. Baseline PCS was not correlated with most post-implant PROMIS-29 domains.

**Conclusion:**

PCS decreased substantially and persistently following SCS implantation among patients with available baseline PCS data. Baseline PCS was not significantly associated with subsequent pain response or most patient-reported outcomes. PCS may represent a dynamic outcome measure that complements conventional pain-intensity assessment.

## Introduction

Chronic pain is one of the most challenging conditions to manage in clinical practice, since it often resists conventional therapies and severely impacts quality of life [1, 9]. Spinal cord stimulation (SCS) is a recognized therapy for selected patients with refractory neuropathic pain, and certain other chronic pain conditions, including complex regional pain syndrome and persistent radicular pain [6, 16, 18]. Its efficacy varies according to the underlying pain mechanism and clinical condition. SCS is believed to modulate ascending pain signals to the brain by delivering electrical impulses to the dorsal columns of the spinal cord.

Evidence regarding the durability of spinal cord stimulation has expanded considerably. Randomized trials and long-term follow-up studies derived from randomized cohorts have reported outcomes extending to 24–36 months across different chronic pain indications and stimulation paradigms, while randomized follow-up data extending to five years are available for complex regional pain syndrome type I [15, 16, 21]. Although these studies establish a growing long-term evidence base for SCS, the magnitude and durability of treatment benefit remain heterogeneous, and robust predictors of outcome remain elusive. Moreover, long-term studies have primarily emphasized pain intensity, disability, health-related quality of life, and device-related outcomes. The longitudinal course of pain catastrophizing and its relationship to multidimensional physical, emotional, and social outcomes after SCS remain less well characterized [1, 2, 12, 16, 18, 22].

According to the International Association for the Study of Pain, pain is both a sensory and an emotional experience, emphasizing the importance of psychosocial factors in how persistent pain is perceived and managed [3, 5, 26]. Pain catastrophizing is defined as a pattern of negative cognitive and emotional responses to actual and anticipated pain [25, 36]. Although both the Numeric Rating Scale (NRS) and Visual Analogue Scale (VAS) are widely used and validated measures of pain intensity, a growing body of evidence indicates that catastrophizing has been associated with both short- and long-term treatment results [29, 33, 36]. Moreover, recent studies suggest that the Pain Catastrophizing Scale (PCS) is most useful when monitored longitudinally after an intervention, capturing dynamic shifts in cognitive-emotional coping rather than being relied upon solely as a static pre-treatment predictor [11, 31].

Pain catastrophizing merits closer investigation in the context of SCS. If catastrophizing indeed amplifies the negative impact of pain, individuals with higher preoperative scores might be at higher risk of poorer coping and less overall improvement. Conversely, patients who achieve meaningful pain relief through SCS may also show a measurable reduction in catastrophizing. Measuring pain catastrophizing both before and after SCS could therefore offer a more nuanced understanding of patient outcomes than relying solely on pain intensity. Given the multifactorial nature of chronic pain, this approach could help refine the assessment of pain therapies and enhance individualized treatment plans.

The primary aim of this study was to determine changes in pain catastrophizing following SCS, potentially capturing aspects of emotional distress and functional status not fully reflected in the Numeric Rating Scale.

The secondary aim was to evaluate long-term clinical outcomes after SCS implantation and to examine whether baseline pain catastrophizing was associated with post-implant outcomes in patients undergoing spinal cord stimulation.

## Methods

### Study design and setting

We performed a retrospective cohort study using prospectively collected data from patients who underwent spinal cord stimulation (SCS) implantation between 2021 and 2025 at the Neurosurgical Department of the University Hospital of Northern Sweden. Eligible participants were ≥ 18 years old and had either chronic neuropathic pain in the upper or lower extremities, chronic low back pain meeting the criteria for Persistent Spinal Pain Syndrome (PSPS) type I or II, or complex regional pain syndrome (CRPS) type I or II.

### Surgical procedure

SCS implantation was carried out in two stages.

1. Stage 1 – Trial lead placement

Percutaneous Octrode™ leads (Abbott Laboratories) were inserted under fluoroscopic guidance.

2. Stage 2 – Pulse generator implantation or lead explantation

After a 7- day trial, patients with ≥ 50% reduction in pain intensity were classified as responders and received a permanent Proclaim™ implantable pulse generator (IPG; Abbott Laboratories). Non-responders had their trial leads removed and were excluded from the study.

During the trial period, patients completed daily digital questionnaires rating pain intensity, mood, activities of daily living, social participation, and sleep. Improvements in any of these domains were noted as additional indicators of a positive response.

All permanently implanted systems were programmed to deliver passive-recharge burst spinal cord stimulation (BurstDR™, Abbott Laboratories, Plano, TX, USA). For each patient, the individual perception threshold was determined as the lowest amplitude at which stimulation was perceived.

The therapeutic amplitude was individualized but did not exceed 60% of the measured perception threshold. This upper limit was consistent with a published passive-recharge burst programming protocol [8]. Lower burst amplitudes have also been associated with lower pain-intensity and pain-catastrophizing scores [20].

Two specialized neuromodulation nurses with extensive SCS experience performed all programming and follow-up adjustments.

### Data collection

Patient- reported outcome measures were collected prospectively and electronically with the Patientkollen™ management platform, which distributed and stored all digital questionnaires across the perioperative and the two-year follow-up period after SCS. Data were collected at baseline and at follow-up intervals of 3, 6, 12, and 24 months post-SCS implantation.

### Outcome measures

#### Primary outcome: pain catastrophizing scale

The primary outcome measure was pain catastrophizing, assessed using the Pain Catastrophizing Scale (PCS). The PCS is a validated 13-item instrument assessing three dimensions of catastrophizing: rumination, magnification, and helplessness [30]. Total scores range from 0 to 52, with higher scores indicating greater levels of pain catastrophizing.

For analyses of change in PCS and associations between baseline PCS and subsequent outcomes, the analytic population was restricted to patients with available baseline PCS measurements. Of the 58 patients included in the overall cohort, 38 had a baseline PCS measurement and therefore constituted the primary PCS analysis population. At the time of database lock, 22 of the 38 patients in the baseline-PCS cohort had completed the 24-month PCS assessment. Of the remaining 16 patients, 10 had not yet reached the 24-month follow-up, 4 had reached the scheduled follow-up but did not complete the questionnaire, and 2 had discontinued active SCS treatment before the 24-month assessment.

#### Secondary outcomes

Pain intensity was assessed using the Numeric Rating Scale (NRS). Changes in NRS pain intensity were used to classify pain response. A reduction of ≥ 30% from baseline was defined as a moderate response and a reduction of ≥ 50% as a high response.

Multidimensional patient-reported health outcomes were assessed using the PROMIS-29 questionnaire, including physical function, anxiety, depression, fatigue, sleep disturbance, ability to participate in social roles, pain interference, and pain intensity. The PROMIS-29 is a well-established and validated instrument for assessing pain-related outcomes and has shown robust reliability and validity in the context of spine-related disorders [23].

### Statistics

Continuous variables are presented as means with standard deviations (SD) or medians with interquartile ranges (IQR), as appropriate, and categorical variables as frequencies and percentages. For analyses involving PCS, patients were categorized according to baseline PCS score as ≤ 15, 16–24, and > 24, corresponding to low, intermediate, and high levels of catastrophizing [30].

Changes from baseline in PCS and secondary patient-reported outcomes were evaluated at 90, 180, 365, and 730 days using paired t-tests among patients with available measurements at both baseline and the respective follow-up time point after SCS implantation. Associations between baseline PCS and post-implant outcomes were assessed using Spearman's rank correlation. Categorical variables, including pain responder rates, were compared using the chi-square test or Fisher's exact test when appropriate. Analysis of variance or the Kruskal–Wallis test was used to compare continuous variables between baseline PCS groups, according to data distribution.

Because follow-up data were not available for all participants at all time points, each longitudinal comparison was based on patients with both baseline and follow-up measurements for the respective outcome. The number of patients contributing to each analysis is reported in the corresponding tables. Missing observations were not imputed.

A two-sided *p*-value < 0.05 was considered statistically significant. All analyses were performed using IBM SPSS Statistics for Mac, Version 26.0 (IBM Corp., Armonk, NY, USA).

### Ethics

Written informed consent was obtained from all participants. The study was conducted in accordance with the ethical principles of the Declaration of Helsinki and was approved by the Swedish Ethical Review Authority (Dnr 2023–01061–01).

## Results

### Patient demographics

Fifty-eight patients were enrolled (22 men, 36 women) with a mean age of 59 (SD 12) years at the time of SCS implantation. Apart from age, which was higher among men, no baseline sex differences were observed in PROMIS-29 domains or PCS (Table 1). Patients were stratified into three PCS groups: ≤ 15 (*n* = 9), 16–24 (*n* = 10), and > 24 (*n* = 19); baseline PCS measurements were unavailable in 20 patients (34.5%) because the PCS questionnaire was not completed at the preoperative assessment. Across the three groups, baseline values were comparable for physical function (*p* = 0.29), fatigue (*p* = 0.06), sleep disturbance (*p* = 0.25), ability to participate in social roles (*p* = 0.55), pain interference (*p* = 0.88) and NRS pain intensity (*p* = 0.08). However, anxiety (*p* = 0.002) and depression (*p* = 0.019) differed significantly, with higher median scores in groups with higher PCS (Table 2).

**Table 1:** Baseline characteristics

Baseline characteristics			
	Men	Women	*P*-value
Number of patients (N)	22	36	
Age (Mean SD)	64.0 (10.6)	56.6 (11.4)	0.019
Physical function (mean SD)	35.0 (7.1)	33.9 (7.2)	0.67
Anxiety (mean SD)	61.4 (11.1)	59.4 (10.6)	0.58
Depression (mean SD)	57.6 (9.4)	59.7 (8.6)	0.49
Fatigue (mean SD)	59.8 (8.7)	58.8 (8.2)	0.72
Sleep disturbance (mean SD)	57.9 (8.7)	58.3 (8.1)	0.89
Ability to participate in social roles (mean SD)	38.3 (6.4)	38.9 (7.5)	0.81
Pain interference (mean SD)	69.7 (6.7)	71.9 (4.9)	0.25
NRS^a^ (mean SD)	7.8 (2.4)	7.9 (1.3)	0.81
PCS^b^ (mean SD)	28.8 (9.2)	21.3 (12.1)	0.06

^a^Numerical Rating Scale

^b^Pain Catastrophizing Scale

**Table 2:** PROMIS-29 in relation to group of PCS before treatment

	^a^PCS 0–15	^a^PCS 16–24	^a^PCS > 24	*P*-value
Physical function (median IQR)	37.9 (31.9–40.6)	35.0 (30.1–38.0)	30.5 (26.6–39.2)	0.29
Anxiety (median IQR)	53.7 (40.3–60.5)	63.4 (45.8–67.3)	67.3 (61.4–71.2)	0.002
Depression (median IQR)	53.9 (45.0–61.4)	62.3 (50.7–66.6)	63.1 (60.1–65.7)	0.019
Fatigue (median IQR)	55.1 (50.9–61.7)	61.7 (53.5–64.6)	63.7 (58.8–64.6)	0.06
Sleep disturbance (median IQR)	54.3 (47.3–60.9)	59.8 (55.2–62.9)	57.9 (54.3–63.8)	0.25
Ability to participate in social roles (median IQR)	38.8 (34.9–46.2)	39.7 (36.5–41.0)	37.3 (35.7–38.8)	0.55
Pain interference (median IQR)	75.6 (65.9–75.6)	75.6 (68.2–75.6)	71.6 (68.0–75.6)	0.88
^b^NRS (median IQR)	8 (6–8)	9.0 (8–9)	9 (7–9)	0.084

^a^Pain Catastrophizing Scale

^b^Numerical Rating Scale

## Outcomes after SCS implantation

### Longitudinal changes in pain catastrophizing

Pain catastrophizing emerged as a dynamic construct that improved significantly following SCS implantation. At baseline, 9 of 38 patients (24%) had PCS scores below 15, consistent with minimal catastrophizing, whereas 19 patients (50%) scored above 24, consistent with severe catastrophizing. By 90 days after SCS implantation, the proportion of patients with minimal catastrophizing had increased: 20 of 35 patients (57%) had PCS scores below 15, while only 5 patients (14%) had scores above 24. At later follow-ups, the proportion of patients with PCS below 15 continued to reflect sustained improvement; 66% at 180 days, 53% at 365 days and 40% at 730 days.

**Fig. 1:**
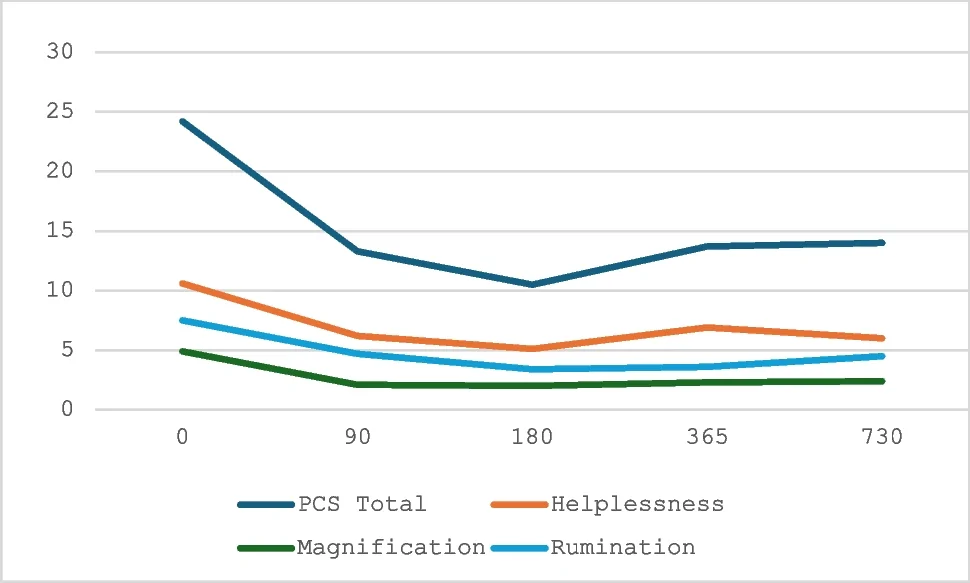
Longitudinal effects of PCS and PCS subgroups after spinal cord stimulation. Mean PCS scores at baseline and at 90, 180, 365, and 730 days after SCS implantation. Mean PCS scores decreased significantly over the follow-up period (*p* < 0.001)

The longitudinal improvement was also evident at the group level. Among patients with available PCS at both baseline and 24 months, mean PCS scores decreased from 24 ± 12 at baseline to 14 ± 11 at the 24-month follow-up (*p* < 0.001; Fig. 1). All three PCS subscales- helplessness, magnification, and rumination showed significant reductions (*p* < 0.01) from baseline, with these improvements maintained throughout the two-year observation period (Table 3).

**Table 3:** The effect on helplessness, magnification and rumination after SCS implantation

	Baseline	90 days	180 days	365 days	730 days
Helplessness, Mean (SD), (n)	10.6 (6.0) (38)	6.2* (5.1) (38)	5.1* (5.2) (33)	6.9* (5.4) (30)	6.0* (4.9) (22)
Magnification, Mean (SD) (n)	4.9 (3.5) (38)	2.1* (2.4) (36)	2.0* (2.5) (32)	2.3* (2.6) (30)	2.4 *(2.5) (22)
Rumination, Mean (SD) (n)	7.5 (4.4) (38)	4.7 *(3.9) (36)	3.4 *(3.7) (32)	3.6* (3.5) (30)	4.5 *(3.7) (22)

*n* = number of patients

^*^p < 0.01

### PCS at baseline and outcomes after SCS implantation

We examined whether baseline PCS scores were correlated with physical function, fatigue, depression, anxiety, sleep disturbance, social-role participation, NRS pain intensity, or pain interference at each post-implant assessment using Spearman’s rank correlation analysis. No significant relationships emerged at any follow-up point except for depression at 730 days follow up (*p* = 0.04) (Table 4). Responder rates were similar across the three baseline PCS groups.

**Table 4:** Spearman’s rank correlation between PCS at baseline and Outcomes after treatment

PCS baseline	90 Days	*P*-value	180 Days	*P*-value	365 Days	*P*-value	730 Days	*P*-value
^a^NRS- Correlation Coefficient	0.05	0.81	0.13	0.56	0.06	0.79	0.27	0.27
Physical function- Correlation Coefficient	−0.01	0.98	0.04	0.87	−0.04	0.87	−0.13	0.58
Anxiety- Correlation Coefficient	0.07	0.74	0.27	0.21	0.27	0.20	0.27	0.20
Depression- Correlation Coefficient	0.20	0.33	0.12	0.58	0.28	0.19	0.46	0.04*
Fatigue- Correlation Coefficient	0.10	0.62	0.28	0.20	0.31	0.15	0.31	0.19
Sleep disturbance- Correlation Coefficient	−0.05	0.80	0.16	0.45	−0.10	0.65	0.45	0.06
Ability to participate in social roles- Correlation Coefficient	−0.04	0.85	−0.03	0.89	0.12	0.57	−0.19	0.43
Pain Interference- Correlation Coefficient	0.19	0.35	0.28	0.19	0.08	0.70	−0.02	0.94

^a^Numerical Rating Scale

## Physical health

Physical function improved significantly following SCS, increasing from a mean of 34.2 ± 7.3 at baseline to 39.2 ± 5.4 at 90 days (*p* < 0.001), with the improvement maintained throughout the 24-month follow-up (all *p* < 0.001). Pain interference was also significantly reduced at all post-implant time points, from 71.7 ± 5.3 at baseline to 62.5 ± 6.3 at 24 months (all *p* < 0.001). Similarly, pain intensity decreased from 7.9 ± 1.7 at baseline to 4.9 ± 1.8 at 90 days and remained significantly lower throughout follow-up (all *p* < 0.001). Fatigue did not differ significantly from baseline at 90, 180, or 365 days, but was significantly reduced at 730 days (*p* = 0.04; Table 5).

**Table 5:** Outcomes after SCS implantation in relation to baseline (Paired t-test)

	Physical function	*p*-value	Anxiety	*p*-value	Depression		Fatigue	*p*-value
Baseline Mean (SD) N	34.2 (7.3) (*n* = 38)	Ref	60.9 (10.2) (*n* = 38)	Ref	59.7 (8.5) (*n* = 36)	Ref	59.8 (8.2) (*n* = 36)	Ref
90 days Mean (SD) N	39.2 (5.4) (*n* = 38)	*p* < 0.001	56.9 (9.7) (*n* = 38)	*p* = 0.003	55.4 (8.4) (*n* = 36)	*P* = 0.004	56.1 (9.9) (*n* = 36)	0.07
180 days Mean (SD) N	39.1 (4.9) (*n* = 34)	*p* < 0.001	55.9 (9.1) (*n* = 35)	*p* < 0.001	54.7 (8.7) (*n* = 32)	*P* = 0.001	56.1 (8.4) (*n* = 33)	0.12
365 days Mean (SD) N	39.3 (6.7) (*n* = 32)	*p* < 0.001	55.2 (9.8) (*n* = 32)	*P* < 0.001	54.9 (9.9) (*n* = 32)	*P* = 0.010	57.0 (9.6) (*n* = 31)	0.15
730 days Mean (SD) N	38.5 (5.2) (*n* = 26)	*p* < 0.001	55.7 (9.1) (*n* = 26)	*P* = 0.017	56.1 (8.0) (*n* = 25)	*P* = 0.001	56.7 (8.2) (*n* = 24)	0.04
	Sleep disturbance		Ability to participate in social roles		Pain interference		^a^NRS	
Baseline	58.0 (8.1) (*n* = 35)	Ref	38.4 (7.3) (*n* = 36)	Ref	71.7 (5.3) (*n* = 36)	Ref	7.9 (1.7) (*n* = 37)	Ref
90 days Mean (SD) N	55.7 (9.6) (*n* = 35)	0.17	44.0 (7.2) (*n* = 36)	*p* < 0.001	60.9 (6.6) (*n* = 36)	*p* < 0.001	4.9 (1.8) (*n* = 37)	*p* < 0.001
180 days Mean (SD) N	55.0 (7.4) (*n* = 33)	0.10	42.3 (5.9) (*n* = 33)	*P* = 0.004	61.5 (5.2) (*n* = 33)	*p* < 0.001	4.7 (1.7) (*n* = 34)	*p* < 0.001
365 days Mean (SD) N	57.0 (8.4) (*n* = 31)	0.57	44.0 (6.9) (*n* = 31)	*p* < 0.001	60.7 (6.2) (*n* = 31)	*p* < 0.001	4.7 (2.3) (*n* = 32)	*p* < 0.001
730 days Mean (SD) N	58.3 (7.1) (*n* = 23)	0.45	41.6 (6.2) (*n* = 25)	*p* = 0.001	62.5 (6.3) (*n* = 24)	*p* < 0.001	5.5 (1.9) (*n* = 24)	*p* < 0.001

^a^Numerical Rating Scale

## Mental health and social function

Anxiety and depression scores decreased significantly following SCS. Anxiety decreased from 60.9 ± 10.2 at baseline to 55.7 ± 9.1 at 24 months (*p* = 0.017), while depression decreased from 59.7 ± 8.5 to 56.1 ± 8.0 (*p* = 0.001). Ability to participate in social roles improved significantly at all post-implant assessments, increasing from 38.4 ± 7.3 at baseline to 41.6 ± 6.2 at 24 months (*p* = 0.001). No significant changes in sleep disturbance were observed during follow-up (Table 5).

## Discussion

In this cohort of patients who had achieved a successful SCS trial and progressed to permanent implantation, baseline PCS was not consistently associated with subsequent post-implantation outcomes. PCS scores nevertheless decreased during follow-up among patients with available data, suggesting that PCS may provide complementary information on cognitive-emotional change after implantation.

SCS is an established treatment option for selected patients with refractory neuropathic pain and certain other pain conditions [1, 7, 13, 14, 32]. The consistent gains across multiple PROMIS 29 domains further underscore the multifaceted nature of chronic pain, which intertwines sensory, emotional, and social processes [3, 5, 11]. Particularly noteworthy were the sustained reductions in depression and anxiety, symptoms strongly linked to pain catastrophizing [19]. Because catastrophizing is thought to magnify the perceived threat value of pain [25], effective neuromodulation may help deprive these negative cognitions of their “emotional fuel”, thereby improving psychological wellbeing.

Previous studies have argued that high preoperative catastrophizing is associated with poorer surgical outcomes [10, 17, 25, 27, 28, 33, 35, 36]. However, such correlation was not evident in the present study. Consistent with earlier work in heterogeneous pain cohorts, we found no association between preoperative PCS scores and either pain-intensity responder status or multidimensional PROMIS domains up to two years post-implantation except for depression at the 2- years evaluation [24, 31]. Our findings support the notion that the PCS behaves as a dynamic construct whose trajectory closely shadows the evolving pain-suffering loop [31]. PCS fell significantly within two years, mirroring sustained reductions in Numeric Rating Scale (NRS) pain intensity, pain interference, and negative affect.

These observations align with the emerging view that catastrophizing may primarily reflect the real-time activity of the medial pathway (emotional components of pain) that transform nociceptive input into suffering [5]. Previous work has also shown that pain catastrophizing decreases following selective nerve root blockade, supporting the potential responsiveness of PCS to changes in pain [31]. Furthermore, emotional domains (anxiety, depression) and social participation improved in tandem with PCS, not merely with the magnitude of analgesia. The sizeable and sustained decline in PCS therefore suggests its responsiveness to SCS-mediated improvements in nociceptive input and cognitive–emotional coping. The decreases across all subscales (helplessness, magnification, rumination) may indicate a broad recalibration of pain appraisal rather than selective dampening of one catastrophizing facet.

In our dataset, PCS appeared to improve earlier and to a larger extent than several PROMIS mental-health domains. This could imply that catastrophic thinking may be the first psychological target to shift when peripheral pain is effectively modulated. Clinically, a falling PCS trajectory might serve as an early surrogate marker of durable benefit long before maximal gains in mood, participation, or function are realized. These results tentatively support adopting an integrated care model combining neuromodulation with targeted psychological support, which may have a potential to maximise SCS benefits [4, 7].

Our findings are consistent with the cohort study by Poulsen et al., which reported no association between pain catastrophizing and SCS outcomes in 259 patients with long-term follow-up [24]. However, these findings differ from multicenter evidence indicating that psychosocial burden, including catastrophizing, may negatively influence SCS trial outcomes [34]. These differences may reflect differences in patient selection, treatment stage, outcome measures, and study design. A key consideration may be how catastrophizing evolves in response to treatment. Serial PCS monitoring offers an inexpensive, rapid barometer of global recovery. A steep early decline signals adaptive cognitive reframing and may predict durable benefit. Conversely, persistently elevated PCS scores, despite acceptable NRS pain relief, should trigger adjunctive psychological interventions such as cognitive-behavioural therapy or mindfulness-based stress reduction to reinforce cortical top-down control.

Incorporating PCS alongside NRS potentially yields a more comprehensive biopsychosocial portrait than intensity ratings alone. PCS changes may precede the full resolution of fatigue, physical function, and social participation, suggesting that it could serve as an early-warning metric when device programming or multimodal care needs adjustment. Notably, our results echo prior multicenter datasets showing that catastrophizing is modifiable and that its amelioration tracks functional restoration, underscoring its suitability as a treatment target rather than a static prognostic label [24].

### Strength and limitations

This study benefits from prospectively collected patient- reported outcomes data and a longitudinal follow-up extending to two-year, confirming that early gains endured, and from the use of validated instruments (PROMIS-29 and PCS), which strengthens internal validity. Nevertheless, several constraints temper generalizability. Foremost is the single-centre design and relatively small sample size, which reduce statistical power and may not represent patients with differing socioeconomic or nociplastic pain profiles. The cohort comprised only patients who achieved at least 50% pain relief during the SCS trial and subsequently underwent permanent implantation. The study therefore cannot determine whether baseline catastrophizing predicts trial success or suitability for SCS. Restriction to trial responders may also have reduced variability in post-implantation outcomes and attenuated associations between baseline PCS and subsequent response. Because the study lacked a control group, the observed changes cannot be attributed specifically to SCS and may partly reflect regression to the mean, expectancy effects, natural variation over time, or unmeasured co-interventions.

Baseline PCS data were missing for 34.5% of participants, introducing potential selection bias and limiting subgroup analyses. Responder categorization relied exclusively on NRS thresholds, incorporating composite endpoints that couple pain relief with functional improvement might have exposed subtler prognostic signals. In addition, PCS was sampled at relatively wide intervals, and denser, ecological momentary assessments could illuminate short-term fluctuations and yield finer-grained predictive models. Finally, we did not control for co-interventions such as cognitive-behavioural therapy, nor did we quantify biopsychosocial modifiers like neuroinflammatory markers, social support, or personal pain beliefs, all factors that might interact with catastrophizing and influence recovery trajectories. Future multicenter studies with larger cohorts, more frequent PCS monitoring, and comprehensive biopsychosocial profiling are needed to validate and extend these findings.

## Conclusion

Among patients with available baseline PCS measurements, pain catastrophizing decreased following SCS implantation and remained lower than baseline among those with available long-term follow-up data. Baseline PCS was not significantly associated with subsequent pain response or most multidimensional patient-reported outcomes. These findings suggest that serial PCS assessment may provide complementary information about the cognitive-emotional dimension of pain beyond conventional pain-intensity measures.

## Data Availability

No datasets were generated or analysed during the current study.

## Declarations

### Authors’ information

The reporting of this observational study complies with the STROBE (Strengthening of Reporting of Observational Studies in Epidemiology) checklist.

## References

[1] Bastiaens F, van de Wijgert IH, Bronkhorst EM, van Roosendaal BWP, van Heteren EPZ, Gilligan C, Staats P, Wegener JT, van Hooff ML, Vissers KCP (2024) Factors predicting clinically relevant pain relief after spinal cord stimulation for patients with chronic low back and/or leg pain: a systematic review with meta-analysis and meta-regression. Neuromodulation 27:70–82 38184342 10.1016/j.neurom.2023.10.188

[2] Bernaerts L, Roelant E, Lecomte F, Moens M, Van Buyten JP, Billet B, Bryon B, Puylaert M, Turgay T, Malone M, Theys T, Van Zundert J, Berquin A, Crombez E, De Coster O, Vangeneugden J, Ly HG, Louagie M, Hans GH (2024) Large-scale real-world data on a multidisciplinary approach to spinal cord stimulation for persistent spinal pain syndromes: first evaluation of the Neuro-Pain® nationwide screening and follow-up interactive register. Front Neurosci 18:1322105 38586192 10.3389/fnins.2024.1322105 PMC10996860

[3] Bustan S, Gonzalez-Roldan AM, Kamping S, Brunner M, Löffler M, Flor H, Anton F (2015) Suffering as an independent component of the experience of pain. Eur J Pain 19:1035–1048 25857478 10.1002/ejp.709

[4] Chakravarthy K, Malayil R, Kirketeig T, Deer T (2019) Burst spinal cord stimulation: a systematic review and pooled analysis of real-world evidence and outcomes data. Pain Med 20:S47–S57 31152177 10.1093/pm/pnz046 PMC6544549

[5] De Ridder D, Adhia D, Vanneste S (2021) The anatomy of pain and suffering in the brain and its clinical implications. Neurosci Biobehav Rev 130:125–146 34411559 10.1016/j.neubiorev.2021.08.013

[6] Deer T, Heros R, Tavel E, Wahezi S, Funk R, Buchanan P, Christopher A, Weisbein J, Gilligan C, Patterson D, Antony A, Ibrahim M, Miller N, Scarfo K, Johnson G, Panchalingam T, Okaro U, Yue J (2024) Comparing conventional medical management to spinal cord stimulation for the treatment of low back pain in a cohort of DISTINCT RCT patients. J Pain Res 17:2741–2752 39193462 10.2147/JPR.S472481 PMC11348985

[7] Deer TR, Mekhail N, Provenzano D, Pope J, Krames E, Leong M, Levy RM, Abejon D, Buchser E, Burton A, Buvanendran A, Candido K, Caraway D, Cousins M, DeJongste M, Diwan S, Eldabe S, Gatzinsky K, Foreman RD, Hayek S, Kim P, Kinfe T, Kloth D, Kumar K, Rizvi S, Lad SP, Liem L, Linderoth B, Mackey S, McDowell G, McRoberts P, Poree L, Prager J, Raso L, Rauck R, Russo M, Simpson B, Slavin K, Staats P, Stanton-Hicks M, Verrills P, Wellington J, Williams K, North R, Neuromodulation Appropriateness Consensus Committee (2014) The appropriate use of neurostimulation of the spinal cord and peripheral nervous system for the treatment of chronic pain and ischemic diseases: the Neuromodulation Appropriateness Consensus Committee. Neuromodulation 17:515–550; discussion 550 10.1111/ner.12208 25112889

[8] Deer TR, Patterson DG, Baksh J, Pope JE, Mehta P, Raza A, Agnesi F, Chakravarthy KV (2021) Novel intermittent dosing burst paradigm in spinal cord stimulation. Neuromodulation 24(3):566–573. 10.1111/ner.13143 32202044 PMC8247280

[9] Falowski SM, Moore GA, Cornidez EG, Hutcheson JK, Candido K, Peña I, Blomme B, Capobianco RA (2021) Improved psychosocial and functional outcomes and reduced opioid usage following burst spinal cord stimulation. Neuromodulation 24:581–590 32583937 10.1111/ner.13226 PMC8247278

[10] Forsythe ME, Dunbar MJ, Hennigar AW, Sullivan MJ, Gross M (2008) Prospective relation between catastrophizing and residual pain following knee arthroplasty: two-year follow-up. Pain Res Manag 13:335–341 18719716 10.1155/2008/730951 PMC2671320

[11] Frumkin MR, Greenberg JK, Boyd P, Javeed S, Shayo B, Shin J, Wilson EA, Zhang JK, Sullivan MJL, Haroutounian S, Rodebaugh TL (2023) Establishing the reliability, validity, and prognostic utility of the Momentary Pain Catastrophizing Scale for use in ecological momentary assessment research. J Pain 24:1423–1433 37019164 10.1016/j.jpain.2023.03.010

[12] Huygen F, Hagedorn JM, Falowski S, Schultz D, Vesper J, Heros RD, Patterson DG, Dehghan S, Ross E, Kyani A, Mansouri MB, Kallewaard JW (2023) Core patient-reported outcome measures for chronic pain patients treated with spinal cord stimulation or dorsal root ganglia stimulation. Health Qual Life Outcomes 21:77 37474950 10.1186/s12955-023-02158-2 PMC10357671

[13] Huygen F, Soulanis K, Rtveladze K, Kamra S, Schlueter M (2024) Spinal cord stimulation vs medical management for chronic back and leg pain: a systematic review and network meta-analysis. JAMA Netw Open 7:e2444608 39541119 10.1001/jamanetworkopen.2024.44608 PMC11565267

[14] Karri J, Orhurhu V, Wahezi S, Tang T, Deer T, Abd-Elsayed A (2020) Comparison of spinal cord stimulation waveforms for treating chronic low back pain: systematic review and meta-analysis. Pain Physician 23:451–460 32967388

[15] Kapural L, Yu C, Doust MW, Gliner BE, Vallejo R, Sitzman BT, Amirdelfan K, Morgan DM, Yearwood TL, Bundschu R, Yang T, Benyamin R, Burgher AH (2016) Comparison of 10-kHz high-frequency and traditional low-frequency spinal cord stimulation for the treatment of chronic back and leg pain: 24-month results from a multicenter, randomized, controlled pivotal trial. Neurosurgery 79(5):667–677. 10.1227/NEU.0000000000001418 27584814 PMC5058646

[16] Kemler MA, de Vet HCW, Barendse GAM, van den Wildenberg FAJM, van Kleef M (2008) Effect of spinal cord stimulation for chronic complex regional pain syndrome type I: five-year final follow-up of patients in a randomized controlled trial. J Neurosurg 108:292–298 18240925 10.3171/JNS/2008/108/2/0292

[17] Kim HJ, Kwon OH, Chang BS, Lee CK, Chun HJ, Yeom JS (2018) Change in pain catastrophizing in patients with lumbar spinal surgery. Spine J 18:115–121 28669860 10.1016/j.spinee.2017.06.028

[18] Kumar K, Taylor RS, Jacques L, Eldabe S, Meglio M, Molet J, Thomson S, O’Callaghan J, Eisenberg E, Milbouw G, Buchser E, Fortini G, Richardson J, North RB (2007) Spinal cord stimulation versus conventional medical management for neuropathic pain: a multicentre randomised controlled trial in patients with failed back surgery syndrome. Pain 132:179–188 17845835 10.1016/j.pain.2007.07.028

[19] Lami MJ, Martinez MP, Miro E, Sanchez AI, Guzman MA (2018) Catastrophizing, acceptance, and coping as mediators between pain and emotional distress and disability in fibromyalgia. J Clin Psychol Med Settings 25:80–92 29450798 10.1007/s10880-018-9543-1

[20] Leong SL, De Ridder D, Deer T, Vanneste S (2021) Potential therapeutic effect of low amplitude burst spinal cord stimulation on pain. Neuromodulation 24(3):574–580. 10.1111/ner.13090 31854070

[21] Mekhail NA, Levy RM, Deer TR, Kapural L, Li S, Amirdelfan K, Pope JE, Hunter CW, Rosen SM, Costandi SJ, Falowski SM, Burgher AH, Gilmore CA, Qureshi FA, Staats PS, Scowcroft J, McJunkin T, Carlson J, Kim CK, Yang MI, Stauss T, Petersen EA, Hagedorn JM, Rauck R, Kallewaard JW, Baranidharan G, Taylor RS, Poree L, Brounstein D, Duarte RV, Gmel GE, Gorman R, Gould I, Hanson E, Karantonis DM, Khurram A, Leitner A, Mugan D, Obradovic M, Ouyang Z, Parker J, Single P, Soliday N (2024) ECAP-controlled closed-loop versus open-loop SCS for the treatment of chronic pain: 36-month results of the EVOKE blinded randomized clinical trial. Reg Anesth Pain Med 49(5):346–354. 10.1136/rapm-2023-104751 37640452 PMC11103285

[22] Odonkor CA, Orman S, Orhurhu V, Stone ME, Ahmed S (2019) Spinal cord stimulation vs conventional therapies for the treatment of chronic low back and leg pain: a systematic review of health care resource utilization and outcomes in the last decade. Pain Med 20:2479–2494 31498396 10.1093/pm/pnz185

[23] Pope JE, Fishman M, Chakravarthy K, Hanes M, Gerling M, Heros R, Falowski S, Shah J, Orhurhu V, Urits I, Deer TR (2021) A retrospective, multicenter, quantitative analysis of patients’ baseline pain quality (PROMIS-29) entering into pain and spine practices in the United States (ALIGN). Pain Ther 10:539–550 33624253 10.1007/s40122-021-00238-z PMC8119505

[24] Poulsen DM, Sørensen JCH, Blichfeldt-Eckhardt MR, Gulisano HA, Knudsen ALH, Nikolajsen L, Meier K (2021) Pain catastrophizing does not predict spinal cord stimulation outcomes: a cohort study of 259 patients with long-term follow-up. Neuromodulation 24:76–85 32929834 10.1111/ner.13213

[25] Quartana PJ, Campbell CM, Edwards RR (2009) Pain catastrophizing: a critical review. Expert Rev Neurother 9:745–758 19402782 10.1586/ERN.09.34 PMC2696024

[26] Raja SN, Carr DB, Cohen M, Finnerup NB, Flor H, Gibson S, Keefe FJ, Mogil JS, Ringkamp M, Sluka KA, Song XJ, Stevens B, Sullivan MD, Tutelman PR, Ushida T, Vader K (2020) The revised International Association for the Study of Pain definition of pain: concepts, challenges, and compromises. Pain 161:1976–1982 32694387 10.1097/j.pain.0000000000001939 PMC7680716

[27] Rhudy JL, Maynard LJ, Russell JL (2007) Does in vivo catastrophizing engage descending modulation of spinal nociception? J Pain 8:325–333 17140855 10.1016/j.jpain.2006.10.003

[28] Riddle DL, Wade JB, Jiranek WA, Kong X (2010) Preoperative pain catastrophizing predicts pain outcome after knee arthroplasty. Clin Orthop Relat Res 468:798–806 19585177 10.1007/s11999-009-0963-y PMC2816776

[29] Springborg AH, Visby L, Kehlet H, Foss NB (2023) Psychological predictors of acute postoperative pain after total knee and hip arthroplasty: a systematic review. Acta Anaesthesiol Scand 67:1322–1337 37400963 10.1111/aas.14301

[30] Sullivan MJL, Bishop SR, Pivik J (1995) The Pain Catastrophizing Scale: development and validation. Psychol Assess 7:524–532. 10.1037/1040-3590.7.4.524

[31] Tabatabaei Shafiei P, Åkerstedt J, Awad A, Sjöberg RL, Wänman J (2025) A prospective study of the association between pain and catastrophizing after selective nerve root blockade. Pain Pract 25:e70017 40035355 10.1111/papr.70017 PMC11877626

[32] Taylor RS, Desai MJ, Rigoard P, Taylor RJ (2014) Predictors of pain relief following spinal cord stimulation in chronic back and leg pain and failed back surgery syndrome: a systematic review and meta-regression analysis. Pain Pract 14:489–505 23834386 10.1111/papr.12095 PMC4238825

[33] Theunissen M, Peters ML, Bruce J, Gramke HF, Marcus MA (2012) Preoperative anxiety and catastrophizing: a systematic review and meta-analysis of the association with chronic postsurgical pain. Clin J Pain 28:819–841 22760489 10.1097/AJP.0b013e31824549d6

[34] Thomson S, Helsen N, Prangnell S, Paroli M, Baranidharan G, Belaïd H, Billet B, Eldabe S, De Carolis G, Demartini L, Gatzinsky K, Kallewaard JW, Winkelmüller M, Huygen F, Stoevelaar H (2022) Patient selection for spinal cord stimulation: the importance of an integrated assessment of clinical and psychosocial factors. Eur J Pain 26(9):1873–1881. 10.1002/ejp.2009 35856311

[35] Veljkovic A, Gagne O, Abuhantash M, Younger ASE, Symes M, Penner MJ, Wing KJ, Syed KA, Lau J (2024) High pain catastrophizing scale predicts lower patient-reported outcome measures in the foot and ankle patient. Foot Ankle Spec 17:501–509 35607813 10.1177/19386400221093865 PMC11667948

[36] Wertli MM, Burgstaller JM, Weiser S, Steurer J, Kofmehl R, Held U (2014) Influence of catastrophizing on treatment outcome in patients with nonspecific low back pain: a systematic review. Spine Phila (Pa 1976) 39:263–273 24253796 10.1097/BRS.0000000000000110

